# Positive antineutrophil cytoplasmic antibody serology in patients with lupus nephritis is associated with distinct histopathologic features on renal biopsy

**DOI:** 10.1016/j.kint.2017.04.029

**Published:** 2017-11

**Authors:** Tabitha Turner-Stokes, Hannah R. Wilson, Massimiliano Morreale, Ana Nunes, Tom Cairns, H. Terence Cook, Charles D. Pusey, Ruth M. Tarzi, Liz Lightstone

**Affiliations:** 1 Imperial College Healthcare NHS Trust Lupus Centre, Hammersmith Hospital, London, UK; 2 Renal and Vascular Inflammation Section, Department of Medicine, Imperial College London, Hammersmith Campus, London, UK; 3 Centre for Complement and Inflammation Research, Department of Medicine, Imperial College London, London, UK

**Keywords:** ANCA, glomerulonephritis, renal pathology, systemic lupus erythematosus

## Abstract

Class IV-S lupus nephritis is often associated with more necrosis and fewer subendothelial immune deposits compared to class IV-G lupus nephritis, suggestive of necrotising glomerular inflammation found in antineutrophil cytoplasmic antibody (ANCA)–associated vasculitis. ANCAs are present in a significant proportion of patients with lupus nephritis. Here we determine whether ANCAs are associated with distinct clinical and histopathologic features of lupus nephritis. Thirty-two ANCA-positive biopsies were compared to 222 ANCA-negative biopsies from patients with lupus nephritis. The majority (82%) of ANCA-positive patients had antimyeloperoxidase antibodies. Class IV-S lupus nephritis and glomerular necrosis were significantly more common (36% vs. 16% and 35% vs. 15%, respectively) and isolated Class V lupus nephritis significantly less common (10% vs. 29%) in the ANCA-positive group. ANCA-positive patients had significantly higher dsDNA titers (335u/ml vs. 52u/ml), significantly lower serum C4 concentrations (0.125g/L vs. 0.15g/L) and significantly higher serum creatinine (130μmol/L vs. 84μmol/L) at the time of biopsy. Hence ANCAs appear to influence the histological pattern of lupus nephritis and are associated with worse baseline renal function and more active lupus serology. There was no significant difference in outcome between groups when matched for severity of disease and treatment using propensity scoring. Thus, further studies are needed to examine whether ANCAs in patients with lupus nephritis have a pathogenic role and whether they are associated with worse renal outcomes or are simply a marker of more severe disease.

The classification of lupus nephritis (LN) was revised with the International Society of Nephrology (ISN)/Renal Pathology Society (RPS) Classification in 2003,^1^ which subdivides Class IV LN into segmental (IV-S) and global (IV-G) subclasses based on whether endocapillary involvement in diffuse proliferative LN is predominantly segmental (involving <50% of the glomerular tuft) or global (involving >50% of the glomerular tuft). Part of the rationale for this subdivision of Class IV LN was based on a study by the Lupus Nephritis Collaborative Study Group, which found that patients with “severe” focal segmental glomerular inflammation (involving >50% of glomeruli in the biopsy specimen) tended to have different histopathologic features on biopsy and worse clinical outcomes, with lower 5-year remission rates and poorer renal survival at 10 years (despite similar baseline clinical parameters and similar treatment).^2^ In that study, diffuse segmental glomerular inflammation was associated with more necrosis and fewer subendothelial immune deposits compared with diffuse global glomerular inflammation. The observed difference in the extent of subendothelial immune deposits between these 2 groups led the authors to suggest that the pathogenic mechanisms mediating segmental as opposed to global glomerular inflammation in diffuse proliferative LN may be distinct.

Class IV LN was therefore divided into Class IV-S (when >50% of involved glomeruli have segmental inflammation) and Class IV-G (when >50% of involved glomeruli have global inflammation) in the revised ISN/RPS Classification 2003. A number of centers have used this revised classification to perform retrospective reviews of their patient cohorts, and some have reported that patients with Class IV-S LN have more necrosis and fewer subendothelial immune deposits compared with those with Class IV-G LN.^3^, ^4^ Controversy exists as to whether there is a difference in the clinical outcome associated with each of these subclasses.^5^

It has been suggested that the more necrotic and pauci-immune glomerular inflammation seen in some patients with Class IV-S LN may be associated with patients having antineutrophil cytoplasmic antibodies (ANCAs).^2^, ^3^, ^4^ However, in many of these studies, ANCA testing at the time of biopsy was not available. ANCAs are more common in patients with systemic lupus erythematosus (SLE) compared with the general population and more common still in those with LN compared with other clinical manifestations of SLE.^6^, ^7^, ^8^ A small number of case series in the literature have looked at ANCA serology in patients with focal and segmental LN characterized by necrosis without prominent subendothelial immune deposits. Some report an association between this histopathologic phenotype and ANCA positivity,^9^, ^10^ but others found no association.^11^, ^12^ None of these studies systematically studied ANCA serology in their cohorts of patients with LN. Hence, as yet, there is no convincing evidence in the literature that ANCA positivity in patients with LN is associated with different histopathologic features of glomerular inflammation.

The aim of this study was to perform a retrospective review of our cohort of patients with LN in order to compare those patients who were ANCA positive (ANCA+ve) at the time of biopsy with those who were ANCA negative (ANCA−ve), with respect to histopathologic features of LN, serologic SLE activity, and renal outcomes. In particular, we wanted to determine whether Class IV-S LN was more common in those patients with positive ANCA serology and whether this was associated with more necrosis and fewer immune deposits, akin to the pauci-immune focal necrotizing glomerulonephritis seen in ANCA-associated vasculitis.

In our center, we screen the majority of our patients with biopsy-confirmed LN for ANCA, and thus we have a large cohort of patients for whom ANCA status at the time of renal biopsy is known. We were therefore able to examine systematically our cohort to determine whether there are particular histopathologic and serologic features of SLE associated with ANCA positivity. This contrasts with existing studies in this field that have tended to report ANCA status for patients with LN who have more necrotic and pauci-immune glomerular inflammation on biopsy, which naturally introduces bias into any assessment of an association of this histopathologic phenotype with positive ANCA serology.

## Results

In this retrospective analysis, a total of 254 biopsy specimens from 203 patients with a histologic diagnosis of LN identified in our hospital renal biopsy database (covering the time period 1997–2013) had ANCA serology assessed within 6 months of biopsy. Thirty-two biopsy specimens were from 29 patients who were ANCA+ve, and this group was compared with the remaining 222 biopsy specimens from 174 patients in our cohort who were confirmed to be ANCA−ve at the time of biopsy. There were no cases of drug-induced lupus.

### Demographic characteristics of the patient population

There was no significant difference between the ANCA+ve and ANCA−ve groups with respect to age at biopsy, male:female ratio, ethnicity, duration of LN, or reason for biopsy (Table 1). This was a young and predominantly female patient population, with a distribution of ethnicity representative of the local population in West London.

**Table 1:** Patient characteristics at the time of renal biopsy

Demographic data	ANCA-positive group (*n* = 32)	ANCA-negative group (*n* = 222)
Median age (range) at biopsy, yr	37.5 (16–78)	34 (16–75)
Median (range) duration of LN, yr	0 (0–24)	0.1 (0–28)
Sex (M:F)	1:4	1:3.5
Ethnicity, %	White: 25	White: 30.6
	Asian: 31.2	Asian: 25.7
	Black: 34.4	Black: 27.5
	Other: 9.3	Other: 16.3
ANCA serology, %	MPO: 82	
	PR3: 7	
	MPO and PR3: 11	
Reason for biopsy, %	First presentation: 56	First presentation: 48
	Flare: 34	Flare: 35
	Failure to respond: 9	Failure to respond: 8
	Routine: 0	Routine: 9

ANCA, antineutrophil cytoplasmic antibody; F, female; LN, lupus nephritis; M, male; MPO, myeloperoxidase; PR3, proteinase 3.

No significant difference between the 2 patient groups for all parameters shown.

### ANCA serology

**Figure 1:**
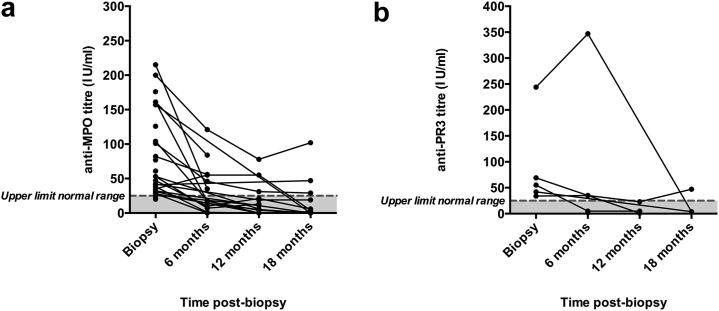
**(a) Anti-MPO and (b) anti-PR3 antibody titers at biopsy and at 6, 12, and 18 months post-biopsy in the ANCA+ve group.** Lines indicate paired data. Normal range indicated by the shaded area. Median anti-MPO and anti-PR3 antibody titers fell to within the normal range at 6 months post-biopsy. ANCA, antineutrophil cytoplasmic antibody; ANCA+ve, antineutrophil cytoplasmic antibody positive; MPO, myeloperoxidase; PR3, proteinase 3.

The majority of patients in the ANCA+ve group had anti-myeloperoxidase (MPO) antibodies (82%) at the time of biopsy, 7% had antiproteinase-3 (PR3) antibodies, and 11% had both anti-MPO and anti-PR3 antibodies (Table 1). In the majority of patients, ANCA antibody titers fell rapidly during the first 6 months post-biopsy (Figure 1), such that the median titers of both anti-MPO (18 IU/ml) and anti-PR3 (20 IU/ml) antibodies were within the normal range by 6 months post-biopsy.

One patient developed increasing anti-PR3 antibody titers during the first 6 months post-biopsy (Figure 1b). This patient had a long-standing history of SLE with multiple disease flares, and her previous treatment included cyclophosphamide and rituximab. ANCA developed at the time of her last renal biopsy when she had had a disease flare with increasing proteinuria and progressive renal impairment. She was poorly adherent to treatment and, hence, unfortunately received suboptimal immunosuppression. She progressed to end-stage renal failure and required hemodialysis 14 months post-biopsy.

### LN has different histopathologic features in patients with ANCA+ve serology compared with those who are ANCA−ve

**Figure 2:**
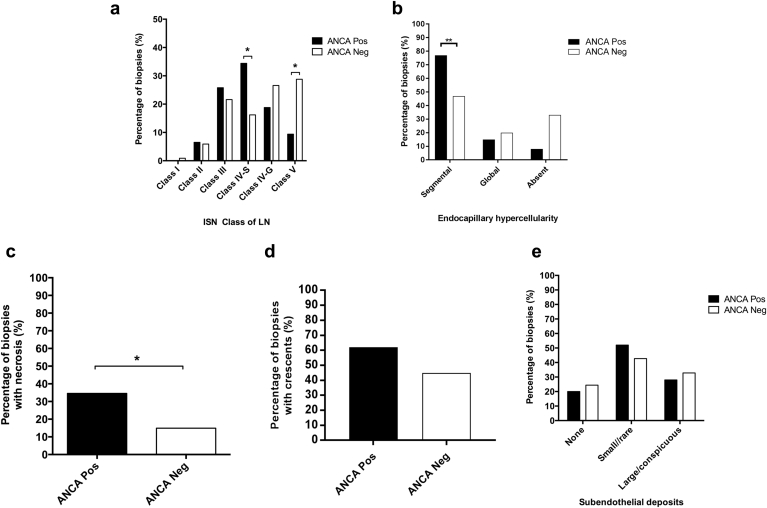
**(a) Percentage of biopsy specimens with each class of lupus nephritis (LN) (according to the International Society of Nephrology/Renal Pathology Society 2003 classification) in the antineutrophil cytoplasmic antibody positive (ANCA+ve) and antineutrophil cytoplasmic antibody negative (ANCA−ve) groups.** Pairwise comparisons demonstrated a significant difference in the proportion of biopsy specimens with Class IV-S and “isolated” Class V (not in combination with other classes) LN in the ANCA+ve compared with the ANCA−ve group. (**b–e**) Include only those biopsy specimens with Class III and IV LN (i.e., proliferative LN) in order to compare the pattern of endocapillary hypercellularity (**b**), the proportion of biopsy specimens with necrosis (**c**), the proportion of biopsy specimens with crescents (**d**), and the extent of subendothelial deposits on electron microscopy (**e**) between the ANCA+ve and ANCA−ve groups. *P < 0.05; **P < 0.01. ISN, International Society of Nephrology; Neg, negative; Pos, positive.

There were significant differences in the histopathologic features of LN in the ANCA+ve group compared with the ANCA−ve group (Figure 2a). A significantly greater proportion of biopsy specimens in the ANCA+ve group had Class IV-S LN (36% vs. 16% in the ANCA−ve group, *P* = 0.0253). Conversely, Class V LN alone (i.e., not in combination with other classes of LN within the same biopsy specimen) was significantly less common in the ANCA+ve group (10% vs. 29%, *P* = 0.0282).

Comparing just those biopsy specimens with proliferative LN (i.e., Class III and Class IV) between the 2 groups, 92% of biopsy specimens in the ANCA+ve group and 67% in the ANCA−ve group had endocapillary hypercellularity (as opposed to scarring). Segmental endocapillary hypercellularity on light microscopy was significantly more common in the ANCA+ve group (77% vs. 47%, *P* = 0.005, Figure 2b). A significantly greater proportion of biopsy specimens in the ANCA+ve group had necrosis (35% vs. 15%, *P* = 0.025, Figure 2c). A greater proportion of biopsy specimens in the ANCA+ve group had crescent formation (62% vs. 45%), but this did not reach statistical significance (*P* = 0.141, Figure 2d). There was no significant difference in the extent of subendothelial electron-dense deposits on electron microscopy (EM) between the 2 groups (*P* = 0.69, Figure 2e).

There was no significant difference in tubulointerstitial inflammation between the ANCA+ve and ANCA−ve groups as assessed by the presence of tubulitis in non-atrophic tubules or the percentage interstitial fibrosis/tubular atrophy. There was also no significant difference in vascular features as assessed by arterial intimal thickening, hyalinosis, or arteritis (Supplementary Table S1).

### Patients with LN and positive ANCA serology have serologically more active SLE

**Figure 3:**
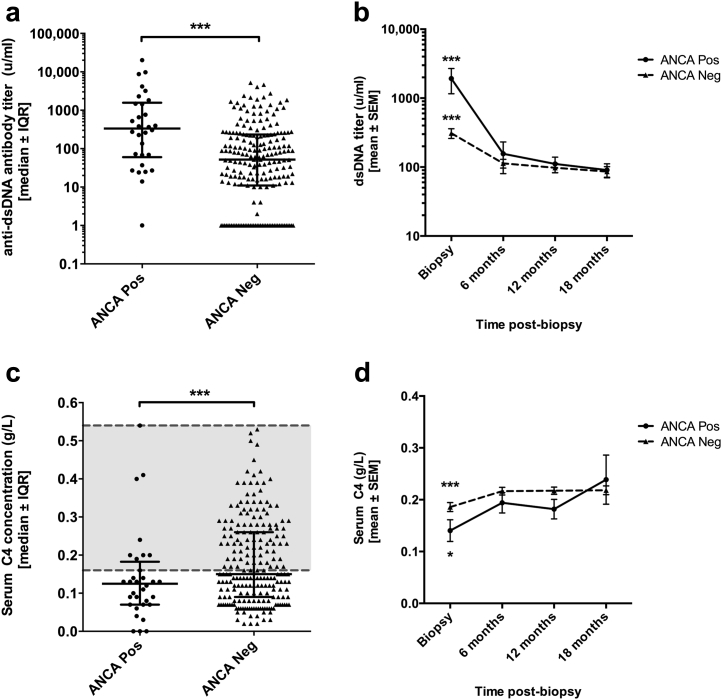
**(a) Anti-dsDNA antibody titers and (c) serum C4 concentration at the time of biopsy were compared between the antineutrophil cytoplasmic antibody positive (ANCA+ve) and antineutrophil cytoplasmic antibody negative (ANCA−ve) groups.** Lines indicate median ± interquartile range (IQR). Data for both parameters were nonparametric in both groups, and comparative analyses were made using a mixed analysis of variance (ANOVA) after transformation of data to achieve normal distribution. Anti–double-stranded DNA (dsDNA) titers were significantly higher and serum C4 concentration significantly lower in the ANCA+ve group compared with the ANCA−ve group. ***P < 0.001. (**b**) Anti-dsDNA antibody titers and (**d**) serum C4 concentrations were also recorded at the 6-, 12-, and 18-month biopsies. Data at all time points were normalized, and a mixed ANOVA was used to compare the change in dsDNA antibody titers over time between the ANCA+ve and ANCA−ve group. Mean ± SEM titers are plotted for each time point. There was a significant decrease in anti-dsDNA titers and increase in serum C4 concentration in both groups during the first 6 months post-biopsy (***significant difference, *P* < 0.001) compared with 6, 12, and 18 months; *P < 0.05 compared with 6 months). There was no significant difference between the 2 groups in either of these parameters at these later time points. Neg, negative; Pos, positive.

Anti–double-stranded DNA (dsDNA) antibody titers and serum C4 concentration were used as serologic markers of SLE activity and compared at the time of biopsy and at 6, 12, and 18 months post-biopsy between the 2 groups. Patients in the ANCA+ve group had significantly higher anti-dsDNA antibody titers at the time of biopsy compared with the ANCA−ve group (median [range]: 335 units/ml [0–20,171] vs. 52 u/ml [0–5200], *P* = 0.00003, Figure 3a). Similarly, serum C4 concentration was significantly lower in the ANCA+ve group at the time of biopsy compared with the ANCA−ve group (median [range]: 0.125 g/l [0–0.54] vs. 0.15 g/l [0.02–1.06], *P* = 0.009) (Figure 3c).

There was a significant improvement in anti-dsDNA titers and serum C4 concentration in both groups during the first 6 months post-biopsy (Figure 3b and d). There were no significant differences between the 2 groups with respect to anti-dsDNA antibody titers or serum C4 concentration at these later time points (Figure 3b and d).

### Renal function and proteinuria

**Figure 4:**
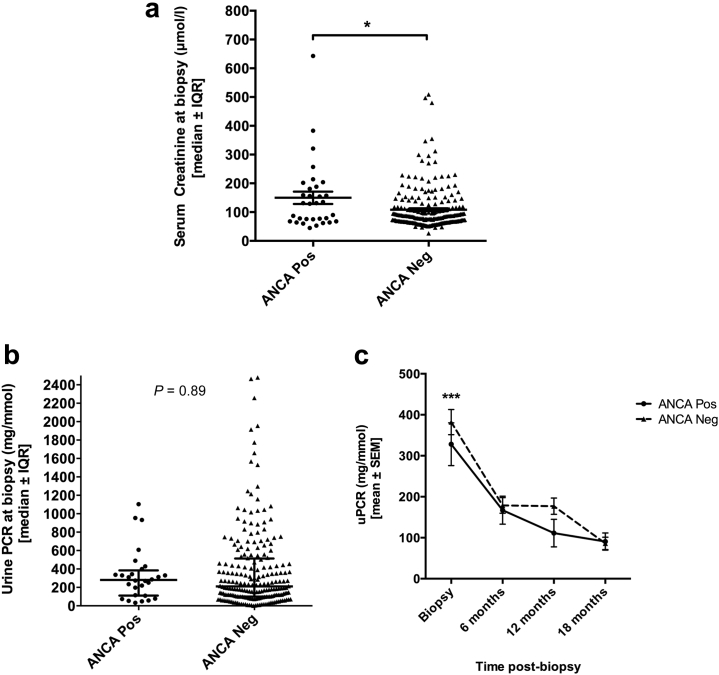
**Serum creatinine level (a) and urine protein:creatinine ratio (uPCR) (b) at the time of biopsy were compared between the antineutrophil cytoplasmic antibody positive (ANCA+ve) and antineutrophil cytoplasmic antibody negative (ANCA−ve) groups.** Lines indicate median ± interquartile range (IQR). Data were nonparametric, and comparative analyses were done using the Mann-Whitney *U* test (serum creatinine) or mixed analysis of variance (ANOVA) after transformation of data to achieve normal distribution (uPCR as repeated measures at different time points compared; see below). *P < 0.05. uPCR was also recorded at 6, 12, and 18 months post-biopsy (**c**). Data at all time points were normalized, and a mixed ANOVA was used to compare the change in uPCR over time between the ANCA+ve and ANCA−ve groups. Mean ± SEM are plotted for each time point. There was a significant decrease in proteinuria overall during the first 6 months post-biopsy (***significant difference [*P* < 0.001] compared with 6, 12, and 18 months). However, there was no significant difference between the ANCA+ve and ANCA−ve groups (*P* = 0.89). Neg, negative; Pos, positive.

Patients in the ANCA+ve group had significantly worse renal impairment at the time of biopsy compared with the ANCA−ve group (median serum creatinine, 130 μmol/l vs. 84 μmol/l, *P* = 0.047). However, both groups contained patients with advanced renal impairment as well as those with relatively normal renal function (range: serum creatinine 45–643 μmol/l in the ANCA+ve group and 26–509 μmol/l in the ANCA−ve group (Figure 4a).

Patients in both groups had significant proteinuria at the time of biopsy (urine protein:creatinine ratio, median [range]: 281 mg/mmol [33–1104] in the ANCA+ve group vs. 210 mg/mmol [0–2480] in the ANCA−ve group), but there was no significant difference in the overall severity of proteinuria between the 2 groups (*P* = 0.89, Figure 4b and c). There was a significant reduction in proteinuria in both groups during the first 6 months post-biopsy (*P* < 0.001, Figure 4c).

### Treatment

**Figure 5:**
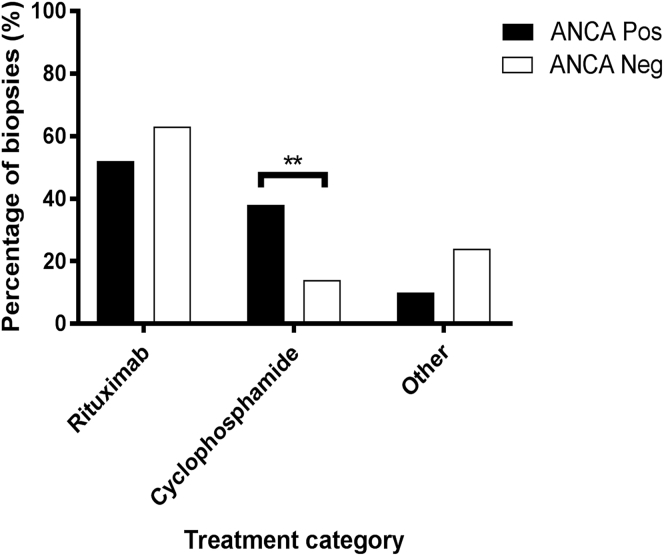
**Column graph demonstrating the distribution of treatment given in response to the renal biopsy in the antineutrophil cytoplasmic antibody positive (ANCA+ve) and antineutrophil cytoplasmic antibody negative (ANCA−ve) groups.** There was a significant difference in the distribution of the treatment, with a higher proportion of patients in the ANCA+ve group receiving cyclophosphamide (*P* = 0.003). **P < 0.01. Neg, negative; Pos, positive.

Treatment protocols in use at our unit are standardized. Patients were treated with one of the following regimens depending on clinical scenario and clinician’s decision: Rituxilup,^13^ consisting of rituximab and mycophenolate mofetil; Rituxirescue,^14^ which consists of the same treatment with continuation of preexisting oral steroids; modified Euro-Lupus^15^; or variable combinations of oral treatments. Rituximab and cyclophosphamide were also used in combination. Where oral steroids were part of the treatment, doses varied. Weaning was actively attempted when clinically appropriate. The Rituxilup regimen (rituximab-based therapeutic regimen) involved no oral steroids. Grouping treatments into rituximab-based, cyclophosphamide-based (with or without rituximab), and other, the distribution of treatment across ANCA+ve and ANCA−ve patients was different with a significantly higher proportion of ANCA+ve patients receiving cyclophosphamide (*P* = 0.003) (Figure 5).

There was no significant difference in the proportion of patients on immunosuppressive treatment immediately before the time of biopsy between the groups.

### Long-term outcomes

**Figure 6:**
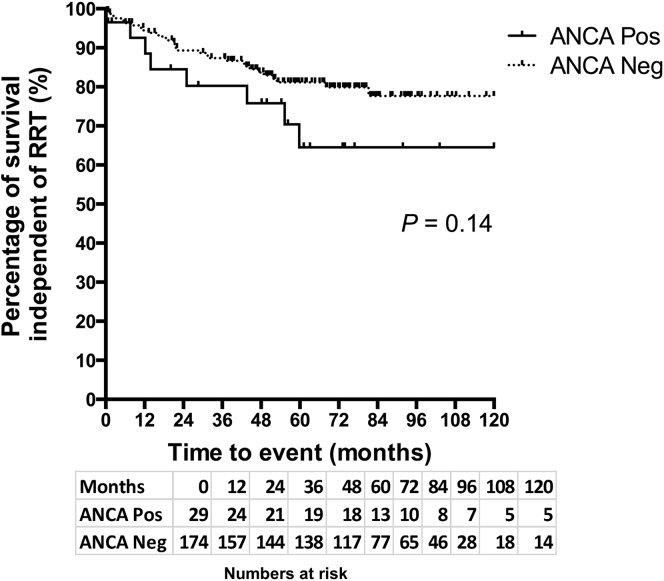
**Kaplan-Meier survival curve illustrating patient survival independent of renal replacement therapy (RRT) in the antineutrophil cytoplasmic antibody (ANCA) positive (Pos) and ANCA negative (Neg) groups (all patients shown).** There was no statistically significant difference in the time to death or RRT between the 2 groups (log-rank test, *P* = 0.14). A Kaplan-Meier survival analysis of propensity-matched groups (2:1 ANCA Neg to ANCA Pos) with a similar risk of poor outcome confirmed no significant difference in time to RRT or death between the 2 groups (log-rank test, *P* = 0.89; graph not shown).

Patient survival independent of renal replacement therapy (RRT) during the post-biopsy period was analyzed (174 ANCA+ve patients and 29 ANCA−ve patients). Overall, the median duration of follow-up was 55 months in the ANCA +ve group and 60 months in the ANCA−ve group. Patient death and commencement of RRT (either dialysis or transplantation) were considered events. Data were censored if patients were lost to follow-up or if an event did not occur by April 10, 2015 when outcomes for all patients were analyzed. There were 8 events in the ANCA+ve group and 32 events in the ANCA−ve group over the entire duration of follow-up. There was no significant difference in time to RRT or death between the 2 unmatched groups, as determined by Kaplan-Meier analysis (log-rank test, *P* = 0.14, Figure 6). In order to compare the outcome of ANCA+ve patients with that of ANCA−ve patients with a similar risk of poor outcome, patients were matched for age, baseline creatinine and proteinuria levels, and treatment using propensity scoring at a 2:1 ratio of ANCA−ve to ANCA+ve. A Kaplan-Meier survival analysis of these matched groups confirmed no significant difference in time to RRT or death between the 2 groups (log-rank test, *P* = 0.89).

## Discussion

This was a retrospective analysis of a large cohort of patients with LN over a long follow-up period (1997–2015) performed to determine whether ANCA+ve serology in patients with LN is associated with different histopathologic features on renal biopsy specimens and different clinical outcomes.

Our results suggest that patients with LN and positive ANCA serology are more likely to have segmental endocapillary hypercellularity on renal biopsy specimens (ISN/RPS Class IV-S LN) with more necrosis and less likely to have isolated Class V LN compared with ANCA−ve patients. Positive ANCA serology at the time of biopsy also appeared to be associated with serologically more active lupus (higher dsDNA titers and lower serum C4 concentration) and worse baseline renal function. There was a similar and significant improvement in anti-dsDNA antibody titers, serum C4 concentration, and proteinuria during the first 18 months post-biopsy in both groups. Our survival analysis suggests that there was no significant difference in the time to death or RRT between the 2 groups. Propensity score matching was performed, which indicated that there was no significant difference in outcome when the groups were matched for factors that affect outcome, thus indicating that ANCA is not an independent risk factor for outcome. However, ANCA was associated with more severe disease in our cohort. It is clearly difficult to draw any firm conclusions on the effect of positive ANCA serology on long-term outcomes in this patient population as this was a retrospective study, and hence treatment with immunosuppressive agents was not standardized and indeed was found to differ between the 2 groups, with a significantly higher proportion of ANCA+ve patients receiving cyclophosphamide. Cyclophosphamide may have been preferentially selected as a treatment in the ANCA+ve group due to the positive ANCA serology or because of the histopathologic features. Cause of death is not known nor whether patients had further flares before requiring RRT. Our findings indicate that further prospective studies are required to assess any effect of positive ANCA serology on outcomes.

Another limitation of this study is the bias in ANCA testing. This was a retrospective analysis, and, although we screened the majority of patients with LN in our cohort for ANCA as part of a renal autoimmune screen, not all patients in our cohort had ANCA testing pre-biopsy. We included patients who had ANCA screening up to 6 months post-biopsy. It is possible that patients were more likely to be screened for ANCA post-biopsy if they had histopathologic features suspicious for ANCA-associated disease (e.g., necrosis). This may be why Class IV-S LN was more common in the ANCA+ve group.

However, to our knowledge, this is the first study that screened a large number of patients with LN for ANCA and looked specifically at whether ANCA positivity is associated with different histopathologic features of LN according to the criteria specified in the revised ISN/RPS Classification 2003. Our data suggest that a significant proportion of patients with LN are ANCA+ve (14% in our cohort), supporting the findings from previous studies that ANCAs are more common in patients with SLE, particularly those with LN, compared with the general population.^8^, ^16^ In our study, the ANCA+ve patients tended to have more serologically active SLE compared with the ANCA−ve patients, a finding that has also been reported by others.^8^, ^16^ This suggests that patients with active lupus may have an enhanced tendency to produce autoantibodies, including ANCAs. Of note, 11% of our cohort were double positive for anti-MPO and anti-PR3 antibodies. We would therefore suggest that all patients with LN be screened for ANCA, and it may be worth considering whether screening for other autoantibodies that mediate glomerular pathology are warranted, including anti-cardiolipin and anti–glomerular basement membrane antibodies.

The different histopathologic features of ANCA+ve patients with LN are interesting and raise the question as to whether the ANCAs in these patients have a pathologic action resulting in different mechanisms of glomerular inflammation, which may lead to worse renal outcomes. In ANCA-associated vasculitis, there is accumulating evidence that ANCAs play a pathogenic role (reviewed in Jennette and Falk^17^ and Tarzi *et al.*^18^). The classic renal manifestation of this disease is a segmental necrotizing crescentic glomerulonephritis, without significant immune deposits (so-called pauci-immune). In our cohort, ANCA+ve patients with LN tended to have a more segmental and necrotizing pattern of glomerular inflammation on renal biopsy compared with those with negative ANCA serology. However, the histology was not pauci-immune, as the extent of subendothelial immune deposits assessed by EM was similar in both groups. This is likely explained by the fact that these patients have immune complexes associated with SLE. In fact, the ANCA+ve patients in our cohort had serologically more active SLE than the ANCA−ve patients. The lower serum C4 concentration in this group suggests that they had increased consumption of complement components by circulating immune complexes. It is therefore not surprising that these patients had evidence of immune complex deposition on renal biopsy rather than a pauci-immune phenotype.

A number of studies have reported different histopathologic features of Class IV-S compared with Class IV-G LN. They have suggested that Class IV-G often appears to be classically immune complex–mediated with wire loop lesions and abundant subendothelial immune deposits correlating with endocapillary hypercellularity, whereas Class IV-S LN is often associated with more necrosis, which can be disproportionate to the extent of subendothelial immune deposits and endocapillary hypercellularity.^2^, ^3^, ^19^, ^20^, ^21^ This may reflect different underlying pathogenetic mechanisms of glomerular inflammation, and some have suggested a role for ANCAs. We believe that our data suggest that there is an association between ANCAs and Class IV LN with more segmental and necrotizing endocapillary inflammation. ANCAs may therefore mediate distinct mechanisms of glomerular inflammation in these patients, superimposed on the effects of immune complex deposition.

Further studies are now needed to examine whether ANCAs in patients with LN have a pathogenic role and mediate glomerular inflammation through distinct mechanisms, such as those found in patients with ANCA-associated vasculitis. This is important to elucidate as a significant proportion of patients with LN are ANCA+ve, and if there are different pathogenetic mechanisms mediating glomerular inflammation in these patients, it is possible that they would respond better to different immunosuppressive regimens targeted at these distinct pathologic processes. We also need further prospective studies with standardized treatment to determine whether long-term outcomes in ANCA+ve patients with LN are worse compared with those who are ANCA−ve, as this will guide appropriate screening, monitoring, and treatment of these patients.

## Methods

This study was a retrospective review of our cohort of patients with LN at the Imperial College Renal and Transplant Centre, Hammersmith Hospital, London. Patients with LN were identified from our hospital renal biopsy database, which included biopsy specimens taken between May 14, 1997 and June 8, 2013. Only those patients who had ANCA serology reported within 6 months of their renal biopsy were included in this study. ANCA+ve serology was defined as an elevated titer of anti-MPO or anti-PR3 antibodies detected by enzyme-linked immunosorbent assay (using the normal range determined by our laboratory). A positive test for ANCA based on indirect immunofluorescence alone was not sufficient to define a patient as ANCA+ve.

Renal biopsy reports were reviewed to determine the following:

● Class of LN, according to the ISN/RPS Classification 2003
● Presence of necrosis
● Presence of crescent formation
● Pattern of endocapillary hypercellularity on light microscopy
● Extent of subendothelial electron-dense deposits on EM, as a marker of immune complex deposition in glomerular capillaries.
● Features of acute and chronic tubulointerstitial changes assessed by the presence of tubulitis in non-atrophic tubules and the percentage interstitial fibrosis/tubular atrophy.
● Features of vascular changes assessed by the presence of arterial intimal thickening, arteriolar hyalinosis, and arteritis.

The reporting of renal biopsies in our unit by our renal histopathologists is standardized to provide a clear description of the features of LN included in the ISN/RPS classification system. The pattern of endocapillary hypercellularity was defined as segmental, global, or absent, as stated in the biopsy report. Subendothelial electron-dense deposits were categorized as either small/rare, large/conspicuous, or absent based on the EM reports, which, in our unit, are reported by a single histopathologist who uses consistent terminology to describe the extent of electron-dense deposits.

The patients’ clinical records were reviewed retrospectively to determine the serum creatinine, estimated glomerular filtration rate, urine protein:creatinine ratio, serum anti-dsDNA antibody titer, and serum C4 concentration at the time of biopsy and at 6, 12, and 18 months post-biopsy. Clinical records were also reviewed for treatment, both in use at the time of biopsy and given in response to the biopsy in question. Survival data were collected retrospectively to determine the patients’ survival independent of RRT between the time of biopsy and April 10, 2015, when all data were censored. Baseline demographic data at the time of biopsy were also collected from review of the patients’ clinical records, including age, sex, ethnicity, reason for biopsy, and duration of LN.

All analyses were performed comparing ANCA+ve biopsy specimens (ANCA+ve group) with ANCA−ve biopsy specimens (ANCA−ve group) except for the outcome analysis, percentage of ANCA positivity, and MPO/PR3 proportion where ANCA+ve patients were compared with ANCA−ve patients. Where there was more than 1 biopsy specimen per patient, the time point of the first biopsy specimen was taken to represent that patient in the outcome analysis.

### Statistical analysis

Data analysis was performed using the IBM SPSS Statistics and GraphPad Prism software programs. Data were assessed for normality of distribution using the Kolmogorov-Smirnov test. Nonparametric data were compared between the ANCA+ve and ANCA−ve groups using the Mann-Whitney *U* test (patient age, duration of LN, and serum creatinine, urine protein:creatinine ratio, anti-dsDNA antibody titers, serum C4 concentration at the time of biopsy, and percentage of interstitial fibrosis/tubular atrophy. The χ^2^ or Fisher exact test was used to compare categorical data between the 2 groups (patient sex and ethnicity, class of LN, reason for biopsy, treatment both in use at the time of biopsy and given in response to the biopsy, endocapillary hypercellularity, glomerular necrosis, crescent formation, extent of subendothelial deposits on EM, tubulitis, arterial intimal thickening, arteriolar hyalinosis, and arteritis). The Fisher exact was used if any value was <5 and in all comparisons in which there were 2 categories. A mixed analysis of variance, after normalization of the data, was used to compare clinical parameters at multiple time points (at biopsy and 6, 12, and 18 months post-biopsy) between the 2 groups (anti-dsDNA antibody titers, serum C4 concentration, urine protein:creatinine ratio). dsDNA titers and urine protein:creatinine ratios were normalized using logarithmic transformations with base 10. C4 concentration was normalized using a square root transformation. Kaplan-Meier survival analysis was used to compare patient survival independent of RRT between the 2 groups. Propensity score matching was performed, matching for age, baseline creatinine and proteinuria, and treatment. Using logistic regression modeling, a propensity score estimating the probability of being ANCA+ve was calculated and used for the matching of ANCA+ve patients with ANCA−ve patients in a 2:1 (negative:positive) ratio with a similar risk of poor outcome. A match tolerance of 0.05 was used.

### Ethical approval

This was a retrospective case notes review meeting the criteria for a service evaluation study and hence did not require approval from a Research Ethics Committee. All patients gave their consent for treatment and received standard care according to our accepted unit protocols.

## Disclosure

All the authors declared no competing interests.

## Supplementary Material

Table S1 Tubulointerstitial and vascular histopathologic features.
