# Supplementary material for: Positive antineutrophil cytoplasmic antibody serology in patients with lupus nephritis is associated with distinct histopathologic features on renal biopsy
Source: Kidney Int. 2017 Nov;92(5):1223–31. doi: 10.1016/j.kint.2017.04.029 (PMC5652376; doi:10.1016/j.kint.2017.04.029)
Supplement: Table S1 — Tubulointerstitial and vascular histopathologic features. [file mmc1.docx]

| **Histopathological data** | **ANCA +ve group**  **(n=32)**  **(%)** | **ANCA –ve group**  **(n=222)**  **(%)** |
| --- | --- | --- |
| Tubulitis |  |  |
| Absent | 69 | 80 |
| Present | 31 | 20 |
| Arteriolar hyalinosis |  |  |
| Absent | 100 | 89 |
| Present | 0 | 11 |
| Arteriolar intimal thickening |  |  |
| Absent | 97 | 95 |
| Present | 3 | 5 |
| Arterial hyalinosis |  |  |
| Absent | 100 | 99 |
| Present | 0 | 1 |
| Arterial intimal thickening |  |  |
| Absent | 59 | 61 |
| Present | 41 | 39 |
| Vasculitis |  |  |
| Absent | 97 | 99 |
| Present | 3 | 1 |

**Supplementary Table 1: Tubulointerstitial and vascular histopathological features**

*No significant difference between the two patient groups for all parameters shown*
